# Similar outcome of femoral neck fractures treated with Pinloc or Hansson Pins: 1-year data from a multicenter randomized clinical study on 439 patients

**DOI:** 10.1080/17453674.2019.1657261

**Published:** 2019-08-27

**Authors:** Kristine Kalland, Henrik Åberg, Anna Berggren, Michael Ullman, Greta Snellman, Kenneth B Jonsson, Torsten Johansson

**Affiliations:** a Department of Orthopedic Surgery, Nyköping Hospital, Nyköping, and Department of Clinical and Experimental Medicine, Linköping University, Linköping;; b Department of Orthopedic Surgery, Institution of Surgical Sciences, Uppsala University, Uppsala;; c Department of Orthopedic Surgery, Falu Hospital, Falun;; d Department of Orthopedics, Sahlgrenska University Hospital, Gothenburg/Mölndal, Institute of Clinical Sciences, Sahlgrenska Academy, University of Gothenburg, Gothenburg;; e Department of Orthopedics, Norrköping, and Department of Clinical and Experimental Medicine, Linköping University, Linköping, Sweden

## Abstract

Background and purpose — There are few reports on the efficiency of the Hansson Pinloc System (Pinloc) for fixation of femoral neck fractures. We compare Pinloc with the commonly used Hansson Pin System in a randomized clinical trial. The primary outcome measure is non-union or avascular necrosis within 2 years. We now report fracture failures and reoperations within the first year.

Patients and methods — Between May 2014 and February 2017, 439 patients were included in the study. They were above 50 years of age and treated for a femoral neck fracture at 9 orthopedic departments in Sweden. They were randomized to either Pinloc or Hansson pins. The fractures were grouped as (a) non-displaced regardless of age, (b) displaced in patients < 70 years, or (c) ≥ 70 years old, but deemed unfit to undergo arthroplasty. Follow-up with radiographs and outpatient visits were at 3 and 12 months. Failure was defined as early displacement/non-union, symptomatic segmental collapse, or deep infection.

Results — 1-year mortality was 11%. Of the 325 undisplaced fractures, 12% (21/169) Pinloc and 13% (20/156) Hansson pin patients had a failure during the first year. The reoperation frequencies were 10% (16/169) and 8% (13/156) respectively. For the 75 patients 50–69 years old with displaced fractures, 11/39 failures occurred in the Pinloc group and 11/36 in the Hansson group, and 8/39 versus 9/36 patients were reoperated. Among those 39 patients ≥ 70 years old, 7/21 failures occurred in the Pinloc group and 4/18 in the Hansson group. Reoperation frequencies were 4/21 for Pinloc and 3/18 for the Hansson pin patients. No statistically significant differences were found in any of the outcomes between the Pinloc and Hansson groups.

Interpretation — We found no advantages with Pinloc regarding failure or reoperation frequencies in this 1-year follow-up.

Internal fixation is the preferred choice for treating undisplaced femoral neck fractures and can also be used for displaced femoral neck fractures depending on age or function. There is still a high revision frequency of approximately 11% in undisplaced femoral neck fractures (Gjertsen et al. 2011) and approximately 27% in patients 55–70 years old with displaced fractures (Bartels et al. 2018). It is therefore important to evaluate new implants designed for better internal fixation.

Pinloc (Figure 1) is a development of the commonly used Hansson pins and represents a new concept. Pinloc consists of 3 cylindrical parallel pins with hooks, connected through a fixed angle interlocking plate. The locking plate is not fixed to the femoral cortex, which allows for compression of the fracture along the femoral neck. Biomechanical laboratory studies with composite bone block have shown greater stiffness, torque at failure, and absorbed total energy at failure when fixed with Pinloc compared with 2 Hansson pins (Brattgjerd et al. 2018). Torsional stability is thought to be beneficial for healing of femoral neck fractures (Ragnarsson and Kärrholm 1992) and could possibly mean less pain for the patient and thus facilitate rehabilitation. Additionally, the lateral plate in the Pinloc implant could reduce local soft tissue irritation compared with the use of protruding pins or screw heads, which has been proposed to contribute to local pain. We have found only 1 clinical Pinloc study: a retrospective study on 40 patients with no control group (Yamamoto et al. 2019).

**Figure 1.**
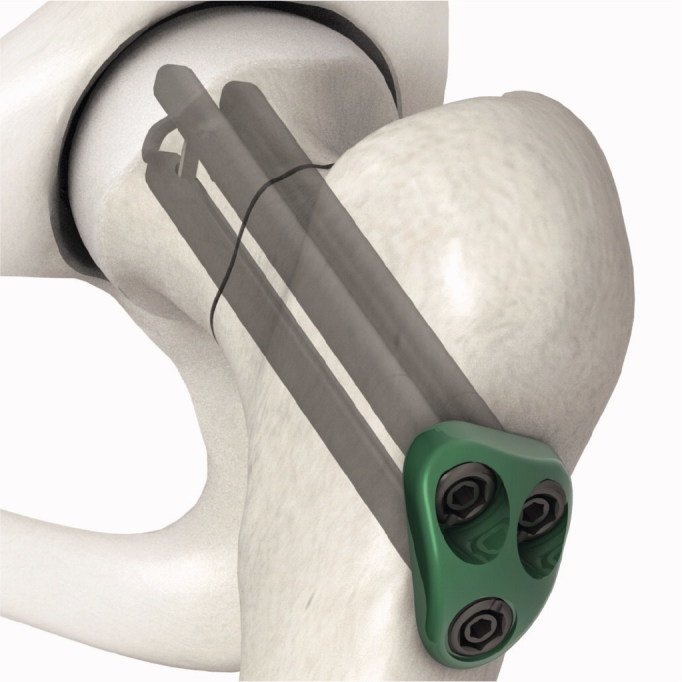
Hansson Pinloc System.

We report fracture failure and subsequent reoperation frequencies at 1 year in the Swedish Pinloc Study, a randomized controlled study comparing Pinloc to 2 standard Hansson pins in the treatment of femoral neck fractures. Failure was defined as deep infection, early displacement, non-union, and symptomatic segmental collapse. The study is designed for 2 years’ follow-up, but we find it ethically important to provide 1-year results. If patients allocated to Pinloc would have a reduced risk of failure or reoperation compared with Hansson pins, it would be wise to implement its use immediately. If not, a widespread introduction of this implant should be avoided while awaiting the final results of 2-year follow-up, including patient-related outcome measures.

## Patients and methods

### Subjects

The study is a prospective randomized controlled trial including participants from 9 orthopedic departments in Sweden. The trial lasted from May 7, 2014 to February 25, 2017. The inclusion of undisplaced fractures ended on September 10, 2016 when 325 patients had been included.

All patients aged 50 years and above, who were admitted to the trial hospitals with a femoral neck fracture considered for internal fixation, were eligible for participation in the study.

**Figure 2.**
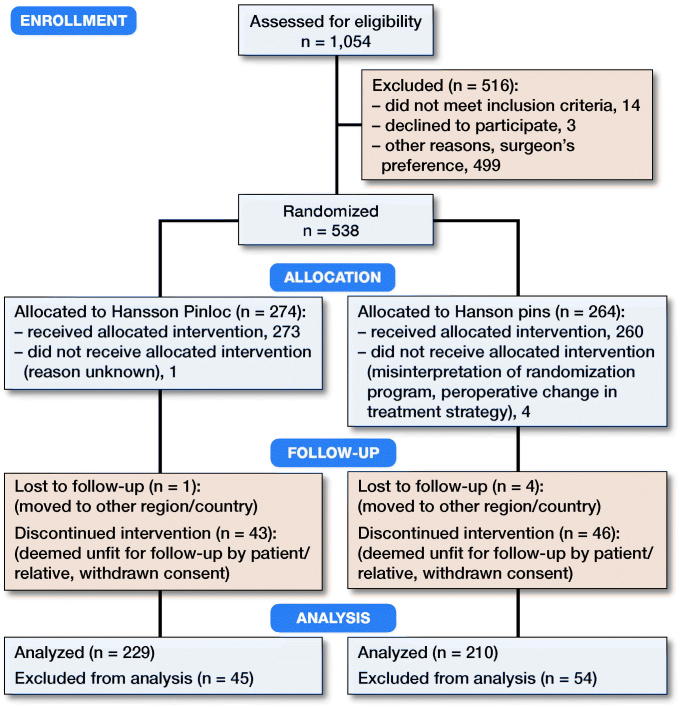
Flow chart of patient enrollment.

At randomization, patients were stratified according to orthopedic department and fracture type: undisplaced/displaced, and if displaced, according to age (Figure 2). Patients with prior inclusion in the study presenting with a fracture in the contralateral hip were not included in the study with the new fracture. The modified Garden classification (Oakes et al. 2003), including lateral view radiographs, was used to classify fractures. Garden I–II fractures were considered undisplaced, whereas Garden III–IV fractures were displaced.

The patients were given oral and written information concerning the trial and provided written or oral consent to participate in the study. In cases of morbidity or mental dysfunction, where the patient was not able to give consent, a proxy (relative or caretaker) granted permission for participation. Patients were randomized in the operating room after fracture reduction, using a digital randomization platform, to receiving either Pinloc or Hansson pins.

### Clinical study protocol

Each hospital had a surgeon in charge of the study and data collection. Anteroposterior and lateral view radiographs of the hip were taken pre- and postoperatively. All patients were allowed full weight-bearing postoperatively.

During the initial hospital admission, information was obtained regarding social conditions, ADL, ASA score, smoking, and use of medications (Table 1), as well as the patient-reported outcome measures (PROMs) WOMAC (Western Ontario and McMaster Universities Osteoarthritis Index), and EQ-5D-3L (EuroQol). Patient follow-up consisted of an outpatient visit including radiographs at 3 and 12 months, PROMs and a TUG test (Timed Up and Go test) (PROMs and TUG data not used in this report). In cases where patients did not attend follow-up appointments, information was obtained either by telephone or by review of patient charts. Failure was defined as early displacement, non-union, avascular necrosis (symptomatic segmental collapse), or deep infection. Reoperation was defined as revision surgery with all causes, except removal of an implant due to local pain, as this is considered a less serious complication. The diagnosis of failure and decision to perform further surgery was made locally, at the discretion of the treating surgeon.

**Table 1.** Demography of patients operated with Pinloc (P) or Hansson pins (H). Values are frequency (percent) unless otherwise stated

Factor	Undisplaced		Displaced age 50–69 years		Displaced age ≥ 70 years	
	P (n = 169)	H (n = 156)	P (n = 39)	H (n = 36)	P (n = 21)	H (n = 18)
Female	129 (76)	115 (74)	19	14	15	11
Male	40 (24)	41 (26)	20	22	6	7
Age, median (IQR)	80 (73–86)	80 (71–87)	59 (56–64)	62 (58–65)	84 (78–87)	82 (77–88)
BMI, mean (SD)	24 (4)	23 (4)	25 (4)	26 (5)	25 (4)	25 (4)
Dementia	31 (18)	19 (12)	0	1	7	5
Smoking	21 (12)	21 (13)	11	13	2	0
Corticosteroids	12 (7)	7 (4)	2	0	1	2

### Statistics

Proportion (chi-square test, Fisher’s exact test) was used to compare deaths, failures, and reoperation frequencies. Power analyses showed that to detect a reduction of failures from 40% to 20% for patients with displaced fractures treated with Pinloc, 64 patients were needed in each group for a power of 80%. As the failure rate of undisplaced fractures is lower and less studied, power analysis was not conducted on failure or reoperation. As we expected a 1-year mortality of 30%, we calculated that 43% more patients needed to be included in the study to reach sufficient numbers of patients. Deceased study persons were included in the analysis until death.

Proportions analyzed with a chi-square test or Fisher’s exact test were used to compare deaths, failures, and reoperation frequencies. P-values < 0.05 were considered to be statistically significant.

### Ethics, registration, funding, and potential conflicts of interest

The study protocol was approved by the regional ethics committee in Linköping 2013-10-16 (dnr 2013/327-31). The study complies with the World Medical Association Declaration of Helsinki Ethical Principles for Medical Research Involving Human Subjects. The study was registered at ClinicalTrials.gov (identifier NCT02776631) and was funded by Region Östergötland. The authors declare no conflicts of interest.

## Results

538 patients were randomized and 439 patients were included in the trial (325 undisplaced and 114 displaced femoral neck fractures) (Figure 2). Patient demographics were similar in the Pinloc and Hansson pin groups (Table 1).

The risk of fracture failure varies greatly between displaced and undisplaced fractures as well as between younger patients and those treated with internal fixation due to medical impairments. For these reasons, the data analyses of these groups are presented separately.

### Undisplaced fractures

No statistically significant difference was found in the failure frequency at 1 year between Pinloc (12%, 21/169), and Hansson pins (13%, 20/156) (Table 2). At the 1-year follow-up, 16/169 patients in the Pinloc group and 13/156 patients in the Hansson group had undergone subsequent surgery (other than extraction of the implant only). The indications for reoperation were similar between groups (Table 3). Implant removal due to local pain was done in 9% (15/169) of Pinloc and 4% (6/156) of Hansson cases respectively (Table 4). 1-year mortality was 12% in both groups.

**Table 2.** Failures and local pain. Values are frequency (percent)

Factor	Undisplaced		Displaced age 50–69 years		Displaced age ≥ 70 years	
	P (n = 169)	H (n = 156)	P (n = 39)	H (n = 36)	P (n = 21)	H (n = 18)
Infection	1 (1)	1 (1)	0	0	0	0
Early displacement/non-union	8 (5)	15 (10)	9	9	4	3
Symptomatic segmental collapse	9 (5)	3 (2)	2	2	3	1
New fracture	3 (2)	1 (1)	0	0	0	0
Local pain	30 (18)	30 (19)	12	17	3	4
Total	51	50	23	28	10	8

**Table 3.** Indication for reoperation. Values are frequency (percent)

Factor	Undisplaced		Displaced age 50–69 years		Displaced age ≥ 70 years	
	P (n = 169)	H (n = 156)	P (n = 39)	H (n = 36)	P (n = 21)	H (n = 18)
Infection	1 (1)	0	0	0	0	0
Early displacement/non-union	6 (4)	10 (6)	7	8	3	3
Symptomatic segmental collapse	6 (4)	2 (1)	1	2	1	0
New fracture	3 (2)	1 (1)	0	0	0	0
Local pain	15 (9)	6 (4)	4	4	1	0
Total	31	19	12	14	5	3

**Table 4.** Type of reoperation. Values are frequency (percent)

Factor	Undisplaced		Displaced age 50–69 years		Displaced age ≥ 70 years	
	P (n = 169)	H (n = 156)	P (n = 39)	H (n = 36)	P (n = 21)	H (n = 18)
THA/HA^a^	11 (7)	12 (8)	8	8	4	3
Re-osteosynthesis^b^	3 (2)	1 (1)	0	0	0	0
Wound revision	1 (1)	0	0	0	0	0
Girdlestone	1 (1)	0	0	1	0	0
Extraction^c^	15 (9)	6 (4)	4	5	1	0
Total	31	19	12	14	5	3

a THA = total hip arthroplasty, HA = hemiarthroplasty

b Re-osteosynthesis: extraction of implant replaced with another internal fixation implant.

c Extraction: removal of implant only (due to pain).

### Displaced fractures, age 50–69

The failure frequency at 1 year was similar in the Pinloc (11/39) and Hansson (11/36) groups. The reoperation frequency and implant removal due to local pain was similar between the groups (Table 3). 1 study person in the Pinloc and 1 in the Hansson group had died at 1 year.

### Displaced fractures, age ≥ 70

Failure frequencies and subsequent surgery were similar between the groups (Table 2 and Table 3). 1/21 Pinloc versus 0/18 Hansson implants were extracted (Table 4). 2 patients in the Pinloc group and 6 in the Hansson group had died at the 1-year follow-up.

## Discussion

We found similar fracture failure and reoperation frequencies in the Pinloc and Hansson groups. Several internal fixation devices for closed reduction and percutaneous fixation have been developed, among them: single nails with a side plate; the sliding hip screw; paired screws or pins; triple screws or pins; and pins with hooks or flanges. However, the results have remained about the same for all designs. Bhandari et al. (2017) compared a sliding hip screw with cancellous screws in the FAITH trial, but found no statistically significant difference in the risk of reoperations. A systematic review of numerous implants for internal fixation of femoral neck fractures showed no statistically significant differences between implants regarding fracture healing complications, reoperations, and mortality (Parker and Gurusamy 2017). This suggests that there are aspects of the healing process of femoral neck fractures that we do not understand.

Different methods and implants may come with specific benefits and risks. The most common implants for internal fixation in Sweden (Hansson pins and Olmed screws) may not offer enough fracture stability, even when optimal reduction and implant position are achieved. Hansson pins are considered easier to use by surgeons and the implant positioning is generally better than for AO screws (Mjørud et al. 2006). Although theoretically appealing, the advantages of Pinloc did not translate into better healing conditions in either undisplaced or displaced fractures in our clinical study. The increased stability of the Pinloc (Brattgjerd et al. 2018), may come at the cost of increased intraosseous pressure caused by the 3 pins in the femoral head. Moreover, the 3 pins connected through the lateral plate may put increased stress on the subtrochanteric region, leading to a higher rate of subtrochanteric fractures. However, these theories were not supported by our study. The frequency of segmental collapse was higher in the Pinloc group, but not statistically significant. This shows that there may not be a clear correlation between biomechanical and clinical studies (Viberg et al. 2017) and highlights the importance of evaluating new implants in randomized clinical trials before general implementation.

This study has several weaknesses. A new device or method has a learning curve. Pinloc is a new concept and the surgical procedure is more demanding for the surgeon than most other implants. Furthermore, in this multicenter study, over 100 different surgeons performed the operations. All had experience with Hansson pins or Olmed screws, but limited practice with Pinloc. Half (499 of 1,047) of the patients who fulfilled the inclusion criteria were never included because the surgeon on call chose not to randomize them. No specific reason had to be given by the surgeon, but common explanations were that the operation would take more time with Pinloc, the surgeon did not feel comfortable with Pinloc, or they simply forgot about the study. The evaluation of radiographs, segmental collapse, early displacement, and non-union may differ between treating surgeons. The threshold for performing revision surgery may also vary between surgeons, hospitals, and implants (Alho et al. 1998). The study was unblinded since the surgeons reviewing radiographs for failures were aware of the type of implant allocated.

### Summary

The preliminary data of the 1-year results from this RCT, show no statistically significant difference in the frequency of failures or reoperations between Pinloc and the Hansson pins in patients over 50 years of age with an undisplaced or a displaced femoral neck fracture. As of today we see no benefit in the use of Pinloc over Hansson pins in femoral neck fractures.
